# Bone mineral density and explanatory factors in children and adults with juvenile dermatomyositis at long term follow-up; a cross sectional study

**DOI:** 10.1186/s12969-021-00543-z

**Published:** 2021-04-26

**Authors:** Henriette Schermacher Marstein, Kristin Godang, Berit Flatø, Ivar Sjaastad, Jens Bollerslev, Helga Sanner

**Affiliations:** 1 Institute for Experimental Medical Research and KG Jebsen Center for Cardiac Research, University of Oslo and Oslo University Hospital, Ullevål, 0027 Oslo, Norway; 2 Bjørknes University College, Oslo, Norway; 3 Section of Specialized Endocrinology, Department of Endocrinology, Morbid Obesity and Preventive Medicine, Medical Clinic, Oslo University Hospital, Oslo, Norway; 4 Institute for Clinical Medicine, Medical Faculty, University of Oslo, Oslo, Norway; 5 Department of Rheumatology, Oslo University Hospital, Rikshospitalet, Oslo, Norway; 6 Department of Cardiology, Oslo University Hospital Ullevål, Oslo, Norway

**Keywords:** Juvenile dermatomyositis, DXA, Bone mineral density, Inflammatory markers, IP-10, Prednisolone

## Abstract

**Background:**

Juvenile dermatomyositis (JDM) is the most common idiopathic inflammatory myopathy in children and adolescents. Both the disease and its treatment with glucocorticoids may negatively impact bone formation. In this study we compare BMD in patients (children/adolescence and adults) with long-standing JDM with matched controls; and in patients, explore how general/disease characteristics and bone turnover markers are associated with BMD*.*

**Methods:**

JDM patients (*n* = 59) were examined median 16.8y (range 6.6–27.0y) after disease onset and compared with 59 age/sex-matched controls. Dual-energy X-ray absorptiometry (DXA) was used to measure BMD of the whole body and lumbar spine (spine) in all participants, and of ultra-distal radius, forearm and total hip in participants ≥20y only. Markers of bone turnover were analysed, and associations with outcomes explored.

**Results:**

Reduced BMD Z-scores (<−1SD) were found in 19 and 29% of patients and 7 and 9% of controls in whole body and spine, respectively (*p*-values < 0.05). BMD and BMD Z-scores for whole body and spine were lower in all patients and for < 20y compared with their respective controls. In participants ≥20y, only BMD and BMD Z-score of forearm were lower in the patients versus controls. In patients, BMD Z-scores for whole body and/or spine were found to correlate negatively with prednisolone use at follow-up (yes/no) (age < 20y), inflammatory markers (age ≥ 20y) and levels of interferon gamma-induced protein 10 (IP-10) (both age groups). In all patients, prednisolone use at follow-up (yes/no) and age ≥ 20y were independent correlates of lower BMD Z-scores for whole body and spine, respectively.

**Conclusion:**

In long-term JDM, children have more impairment of BMD than adults in spine and whole-body. Associations with BMD were found for both prednisolone and inflammatory markers, and a novel association was discovered with the biomarker of JDM activity, IP-10.

**Supplementary Information:**

The online version contains supplementary material available at 10.1186/s12969-021-00543-z.

## Background

Juvenile dermatomyositis (JDM) is the most common idiopathic inflammatory myopathy in children and adolescents. Disease activity is mitigated by immunosuppressing agents used as first-line medication; this includes prednisolone, a glucocortocoid that affects childhood bone modelling and adult bone remodelling negatively [1]. Importantly, in JDM, glucocortocoids dosage [2], treatment duration [3] and age at medication initiation [2] significantly affect bone loss. Optimal skeletal development during childhood and puberty, including bone maturation, is important in order to reach peak bone mass and prevent osteoporosis in adult life [4].

Bone mineral density (BMD) is an estimate of bone mass and is measured by dual-energy X-ray absorptiometry (DXA). However, bone formation, mineralisation and strength are also influenced by inflammatory factors, hormones and the presence of minerals, such as phosphate [5], calcium, 25-hydroxyvitamin D (25(OH)VitD) and parathyroid hormone (PTH) [6]. These markers of bone and mineral metabolism regulate bone modelling (in children) and -remodelling (in adults) by interfering with osteoblast and osteoclast activity and function [7]. Blood serum biomarkers for bone turnover are used in both the daily clinic and in biomedical research [8]. Procollagen type 1 N-terminal propeptide (P1NP), and C-terminal telopeptide of type 1 collagen (CTX) are biomarkers of bone loss in systemic lupus erythematosus (SLE) [9], rheumatoid arthritis [10] and dermatomyositis [11]. However, P1NP and CTX in relation to bone mass and disease activity, to our knowledge, have not been studied in JDM.

Low BMD has been reported in children with JDM, whether or not they were medicated with immunosuppressing treatment [12–15]. There are concerns that such children will not reach peak bone mass, and thus will be at risk of developing osteoporotic fractures later in life [4, 12, 16].

We and others have shown that osteoporosis (included in myositis damage index (MDI)-skeletal domain) is present in 5–9% of JDM patients in the Nordic region after long-term follow-up [17, 18], and in 6% of patients globally [19].

Importantly, we are not aware of any reports evaluating DXA-based BMD in both adolescent and adult JDM patients concurrently. Thus our aim was to evaluate bone mass status and how bone remodelling factors is associated with disease outcomes and prednisolone dosages. We study both children/adolescents and adults included in our unique cohort of Norwegian JDM patients assessed after long-term follow-up compared to controls.

## Patients and methods

### Patients and controls

This study is part of a larger controlled, cross-sectional study in Norway. Patients were included based on a probable or definitive diagnosis of JDM according to the *Bohan and Peter* criteria [20]. Sixty-seven patients diagnosed between January 1970 and June 2006, fullfilled the inclusion criteria: age < 18 years at disease onset, disease duration 24 month and age ≥ 6y at inclusion. These patients were tracked through the National Population Register, and of them, 59 (95%) participated in the overall study [21]. Age- and sex-matched controls were drawn from the same register. Data on controls, including demographics, have previously been published [21].

### Data collection and clinical measurements

During a one- to two-day follow-up programme (September 2005–May 2009), study participants were examined at Oslo University Hospital (OUS). Clinical examination was performed by a single physician (HS), and DXA scans and non-fasting blood samples (serum) were taken and frozen in smaller batches at − 80 °C for later analyses [21]. Disease activity was measured by the JDM disease activity score (DAS)(0–20) [21, 22] and cumulative organ damage by MDI(0–40) [17, 22]. MDI osteoporosis was defined as occurrences of low-energy fracture or vertebral collapse (excluding avascular necrosis). Disease duration was defined as the time from the first muscle or skin symptom associated with JDM to the follow-up examination. The patients’ medication histories, including prednisolone doses, were obtained from the medical records and cumulative doses were calculated by chart review [17]. 87% of patients were treated with oral prednisolone and 15% with intravenous methylprednisolone during disease course; at follow-up 17% were on oral prednisolone [17] .

Participants ≥20y reported average weekly physical activity the last year by self-reporting questionnaire [23]. Due to a low number of replies from participants < 20y they were not included. We categorised sweat-inducing or breathless activities as number of hours of exercise: < or ≥ 2 h/week; and to exercise frequency: < or ≥ 2 activities/week.

In study participants ≥20y, a physical component summary scale (PCS) was measured through use of the Norwegian version of the Short Form 36 health survey (SF-36), version 1.0 [17]. Low scores indicated poor physical status.

### Bone mass measurements

Bone mineral content was determined using DXA. A narrow fan beam densitometer (GE Healthcare Lunar Prodigy, Madison, WI, USA) was used and all the scans were reanalysed in the same software version 14.10 according to a standard protocol. No hardware was changed during the study period. We analysed the anterior-posterior lumbar spine L2-L4 (spine), and whole body (WB) in study participants < 20y using pediatric software and calculated BMD (bone mineral content in g/cm^2^) for these regions. For study participants ≥20y, additionally BMD for ultra-distal and distal 33% radius (forearm), and bilateral proximal femur, dual total hip were analysed using adult software. Hence the participants were divided into two age-groups: younger than 20 years (< 20y), and older or equal to 20 years (≥20y). BMD Z-scores were estimated by comparison with the Lunar reference database incorporated in the software and provided by the manufacturer. The database includes BMD data from healthy subjects in the general population of the United States, which has been validated as applicable for clinical use in the adult [24] as well as the pediatric Norwegian population [25]. Z-scores <−1SD below the age-specific mean for healthy individuals were defined as reduced [12, 26, 27].

### Laboratory analyses

The biomarkers of bone resorption (CTX) and formation (P1NP) were measured by electroluminescence technology using a Cobas e601 (Roche). The bone metabolism marker 25(OH) VitD was measured using liquid chromatography/tandem mass spectrometry. Insufficient amounts of 25(OH) VitD were defined as levels ≤20 nmol/l according to expert consensus [28] and low as ≤37 nmol/l (corresponding to the lower level of the reference value for the general population in Norway). All analyses were performed at the Hormone Laboratory, Department of Medical Biochemistry, OUS, Oslo in 2019. Levels of IP-10 (CXCL10), a known marker of disease activity in JDM [29], was measured as part of a 27-plex cytokine panel (Bio-Plex immunoassay systems, #m500kcaf0y, Bio-Rad, Hercules, CA), based on xMAP technology (Luminex, Austin, TX) in 2012. All samples were handled and analysed according to manufacturer protocol without freeze/thaw cycles. Measurements of erythrocyte sedimentation rate (ESR) and serum levels of high-sensitivity C-reactive protein (hsCRP), and of the bone minerals albumin, phosphate, PTH, alkaline phosphatase and ionised calcium, were performed consecutively at the Department of Medical Biochemistry, OUS or at local hospitals when appropriate. All analyses were performed in non-fasting serum samples according to standard protocols for the analytical methods. We are aware that non-fasting samples contribute to some uncertainty in the absolute measures reported, as feeding affects concentrations of most biological serum factors.

### Statistics

SPSS version 27 (SPSS, Chicago, IL) was used for statistical analyses. Paired samples t-tests, Mann-Whitney U tests, Wilcoxon signed rank and chi-square tests were used to compare patient characteristics, BMD, BMD Z-scores and bone-turnover markers to matched controls. To identify explanatory risk factors for impaired bone density, associations between bone density measures (BMD and BMD Z-scores), markers of bone and mineral metabolism and disease activity measures at follow-up, were analysed using Spearman’s correlation coefficient (rsp) for continuous and Pearson’s correlation coefficient for categorical variables (point bi-serial correlation). Subsequent multivariate linear regression analysis was used in order to identify correlates, with forward selection of possible correlates. Due to the relatively low n, limitations for numbers of variables into the regression analysis forced us to select possible explanatory variables (e.g only one inflammatory marker / prednisone variable). Explanatory variables were included in the models if they showed an association with the outcome variable in univariate analyses or were known from the literature to be associated with the outcomes (*p*-value < 0.1). Due to the hypothesis-generating nature of our study, data was not corrected for multiple comparisons.

## Results

### Characteristics of JDM patients

In total 28/59 (47.5%) of both patients and controls were under the age of 20; 20 girls and eight boys (Table 1). Median disease duration was 16.8y (Table 1); 6.5y (IQR 4.9–8.7y) and 26.3y (IQR 18.9–31.0y) for patients < 20y and > 20y respectively. There was no significant difference in body mass index between patients and controls; however, patients were on average 2.4 cm shorter than controls (*p*-value =0.05). Time from diagnosis to immunosuppressive medication was comparable for both age groups. The occurrence of fractures during life was comparable between patients and controls (Table 1). As previously described, the SF-36 physical component score chart revealed a significantly reduced score for physical activity among the patients compared with controls [23]. The SF-36 scores were 4.3 units lower in patients ≥20y compared with controls. There were no significant differences in neither hours spent nor frequency of physical activity per week in patients vs. controls ≥20y.

**Table 1:** Characteristics and disease variables in JDM patients and controls

	All participants		Age < 20y		Age ≥ 20y	
	Patients (***n*** = 59)^a^	Controls (***n*** = 59)^a^	Patients (***n*** = 28)	Controls ***n*** = 28	Patients (***n*** = 31)	Controls ***n*** = 31
Age, y, median (range)	21.5 (6.7–55.4)	21.6 (6.2–55.4)	15.3 (6.7–19.8)	14.4 (6.2–20.1)	34.3 (20.4–55.4)	34.2 (20.5–55.4)
Female, n (%)	36 (61)	36 (61)	20 (71.4)	20 (71.4)	16 (51.6)	16 (51.6)
BMI, kg/m^2^, mean (SD)	22.3 (4.8)	22.7 (4.5)	NA	NA	24.0 (4.4)	23.8 (3.5)
Weight, kg, mean (SD)	62.6 (20.1)	65.5 (20.1)	52.0 (19.5)	57.0 (22.7)	72.1 (15.7)	73.2 (13.6)
Height, cm, mean (SD)	164.9 (14.7)†	167.3 (15.8)	157.1 (15.9)	159.8 (18.3)	171.8 (9.1)	174.0 (9.2)
Disease duration, y, median (IQR)	16.8 (6.6–27.0)	NA	6.5 (4.9–8.7)	NA	26.3 (18.9–31.0)	NA
Time from diagnosis to medication, month, median (IQR)	4 (2.0–6.25)	NA	4 (1.8–6.3)	NA	4 (2.0–7.5)	NA
Fracture any, n (%)	19 (32.2)	21 (36.8)	7 (25.0)	7 (25.0)	21 (67.7)	14 (48.3)
PRINTO inactive, n (%)	21 (35.6)	NA	9 (32.1)	NA	12 (38.7)	NA
DAS (0–20) at follow up, median (IQR)	5 (3.0–6.0)	NA	5 (2.2–6.0)	NA	4.5 (3.0–7.0)	NA
DAS at diagnosis (0–20), median (IQR)	13.0 (9.0–15.0)	NA	13.0 (9.6–15.0)	NA	13.0 (9.0–16.0)	NA
MDI total (0–40) at follow up, median (IQR)	3 (2–6)	NA	2 (1–4)	NA	5 (3–8)	NA
MDI osteoporosis, n (%)	5 (8.5)	NA	2 (7.1)	NA	3 (9.7)	NA
Smoking daily, n (%)	11 (18.6)	8 (13.6)	3 (10.7)	2 (7.1)	8 (13.6)	6 (19.1)
SF 36 physical component score ≥ 20y, median (IQR)	NA	NA	NA	NA	52.2 (42.4–57.7)†	56.5 (52.8–59.6)
Exercise ≥2 h/week ≥20y, n (%)	NA	NA	NA	NA	19 (61.3)	20 (64.5)
Exercise ≥2 activities/week ≥20 y, n (%)	NA	NA	NA	NA	14 (45.2)	19 (61.3)

*BMI* body mass index, *DAS* disease activity score, *MDI* myositis damage index, *RINTO* paediatric rheumatology international trials organisation, *NA* not applicable, ^a^In each age group *n* = 59 otherwise stated as *n* ≥ 20y = 31 and *n* < 20y =28). †*p* < 0.05. *p*-values when comparing patients and controls using paired sample (2-tailed) or chi-square tests when appropriate

### Bone mass measures in study participants

BMD and BMD Z-scores for WB and spine in all patients tended to be lower than in controls (Table 2). Patients < 20y had lower BMD WB and spine (Δ = 0.04 g/cm^2^ and 0.08 g/cm^2^, *p*-values = 0.02 and 0.04) and BMD Z-scores for WB and spine (Δ = 0.04 g/cm^2^ and 0.08 g/cm^2^, *p*-values = 0.02 and 0.04) compared with controls <20y. However, these differences in BMD and BMD Z-scores for WB and spine were not found between patients and controls ≥20y. For DXA variables that were only assessed in study participants ≥20y, patients had lower BMD and BMD Z-scores for the forearm compared with controls (Δ = 0.06 g/cm^2^ and 0.71 both *p*-values =0.01), whereas no significant differences were found between groups for ultra-distal radius and hip. In study participants ≥20y, there were no differences in T-scores for any of the acquisition regions.

**Table 2:** BMD and Z scores in JDM patients and controls in total, <20y and ≥ 20y

Variable	Patients (***n*** = 59)	Controls (***n*** = 59)	Patients <20y (***n*** = 28) ≥20y (***n*** = 31)	Controls <20y (***n*** = 28) ≥20y (***n*** = 30–31)	***p***-values
**BMD, g/cm**^**2**^					
**Whole body**	1.10 (0.15)	1.13 (0.14)			**0.05**
Age < 20y			1.01 (0.13)	1.06 (0.16)	**0.02**
Age ≥ 20y			1.18 (0.10)	1.19 (0.08)	0.71
**Lumbar spine, L2-L4**	1.12 (0.23)	1.17 (0.22)			0.07
Age < 20y			0.99 (0.21)	1.07 (0.27)	**0.04**
Age ≥ 20y			1.24 (0.18)	1.26 (0.11)	0.52
**Total hip**					
Age ≥ 20y			1.00 (0.18)	1.05 (0.14)	0.10
**Ultra distal radius**					
Age ≥ 20y			0.48 (0.08)	0.5 (0.08)	0.13
**Forearm**					
Age ≥ 20y			0.87 (0.09)	0.93 (0.11)	**0.01**
**Z-score**					
**Whole body**	−0.07 (1.08)	0.27 (0.90)			0.06
Age < 20y			−0.39 (0.99)	0.28 (1.01)	**0.01**
Age ≥ 20y			0.21 (1.10)	0.26 (0.71)	0.83
**Lumbar spine, L2-L4**	−0.16 (1.2)	0.4 (1.02)			0.07
Age < 20y			−0.39 (1.01)	0.25 (1.21)	**0.04**
Age ≥ 20y			0.06 (1.34)	0.24 (0.84)	0.56
**Total hip**					
Age ≥ 20y			−0.32 (1.35)	0.14 (0.99)	0.10
**Ultra distal radius**					
Age ≥ 20y			−0.42 (1.36)	0.16 (1.33)	0.13
**Forearm**					
Age ≥ 20y			−0.76 (1.03)	−0.05 (0.87)	**0.01**
**T-score**					
≥ **20y**					
**Whole body**	NA	NA	0.16 (1.25)	0.29 (0.82)	0.81
**Lumbar spine, L2-L4**	NA	NA	0.00 (1.44)	0.25 (0.92)	0.09
**Total hip**	NA	NA	0.42 (1.41)	0.07 (1.04)	0.07
**Ultra distal radius**	NA	NA	−0.43 (1.35)	0.14 (1.35)	0.48
**Forearm**	NA	NA	−0.77 (1.00)	−0.07 (0.87)	0.38

*BMD* bone mineral density, *y* years. Values for total hip, ultra distal and forearm are not applicable for age < 20y. Values are mean (SD). *p*-values when comparing patients and controls using paired samples t-test

### Occurrence of reduced BMD Z-scores in study participants

**Fig. 1:**
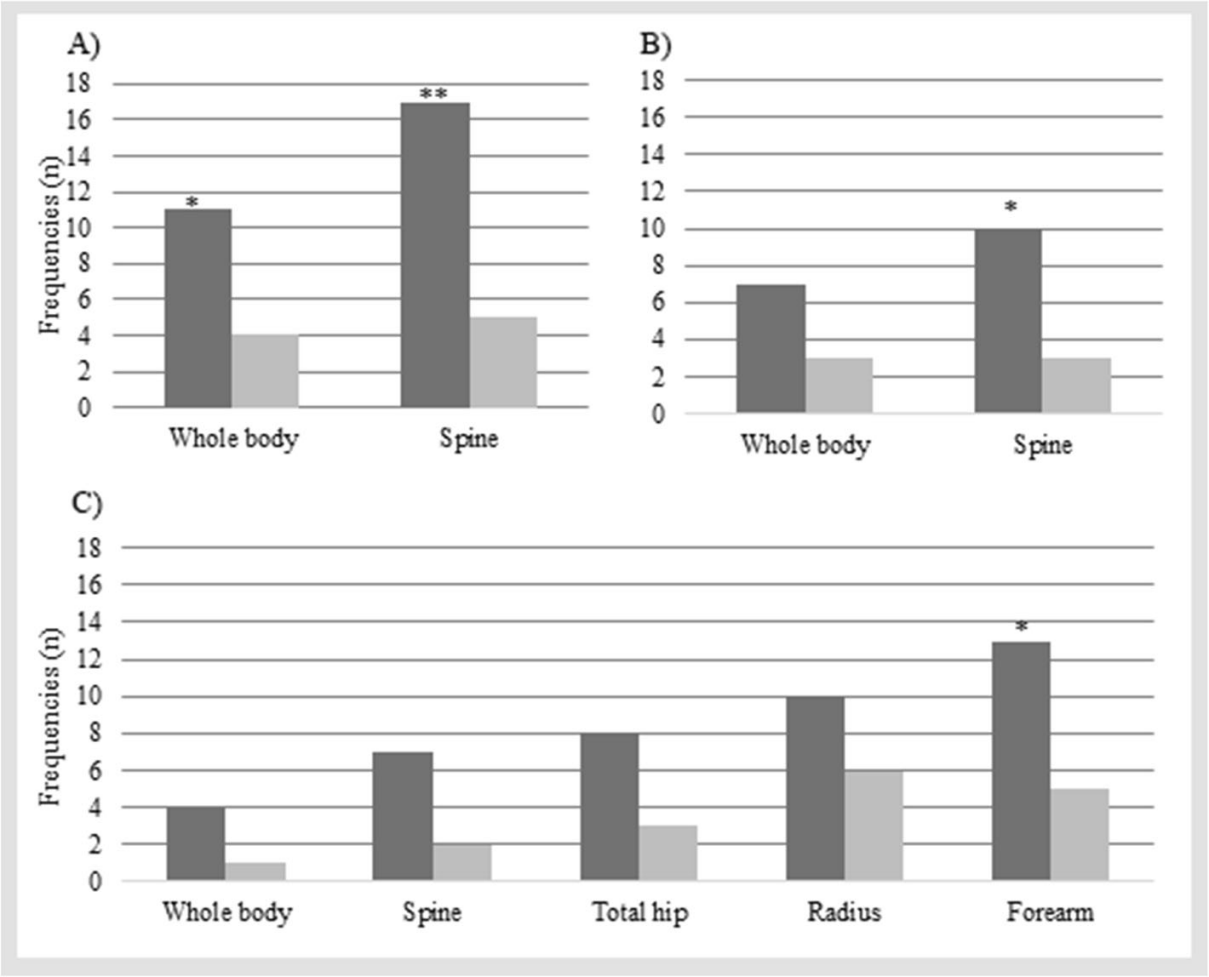
Frequencies of reduced BMD Z-scores in patients compared to controls. (**a**) shows data of WB and lumbar spine in all study participants, (**b**) shows data of WB and lumbar spine in study participants <20y and (**c**) shows data on WB, lumbar spine, total hip, ultra-distal radius and forearm in study participants in study participants ≥20y. WB: WB, LS: lumbar spine, TH: Total hip, UD: Ultra distal radius, FA: Forearm.* *p* < 0.05 and ** *p* < 0.01

12% more patient had reduced BMD Z-scores for WB and 20% more had reduced BMD Z-scores for spine compared to controls (both *p*-values < 0.01) (Fig. 1a). Also, in the participants <20y, 26% more patients than controls had reduced Z-values for the spine (Fig. 1b). In the participants ≥20y, reduced Z-scores in the forearm were found in 26% more patients than in controls; for the other regions examined, there were non-significant trends of more frequent reduced BMD Z-scores for spine and total hip in patients vs. controls (Fig. 1c).

### Prednisolone use in patients

At follow-up, 10 patients (16.9%) used prednisolone; of those, seven were < 20y (Table 3). Two patients <20y and six ≥20y had not been medicated with prednisolone during the disease course. Not surprisingly, patients ≥20y had used prednisolone for twice as long as patients <20y, and cumulatively, their doses were 1.5 times greater than the doses given to patients <20y (both *p*-values =0.02). However, months from last prednisolone dose to FU were 3.4 times longer in patients ≥20y compared to patients <20y. Cumulative prednisolone doses after 6 months and 2 years were comparable between patients <20y and ≥ 20y. However, when comparing cumulative prednisolone doses at 6.5y, (which was the mean time of disease duration for patients <20y), patients ≥20y were medicated with prednisolone doses that on average were 1.5 times as high as those given to patients <20y.

**Table 3:** Medication in JDM patients total, < 20y and ≥ 20y

Medication	Patients (***n*** = 59)	Patients < 20 y (***n*** = 28)	Patients ≥ 20 y (***n*** = 31)	***p***-values
Prednisolone or DMARD at FU, n	17 (28.8)	13 (46.4)	4 (12.9)	**0.004**
Prednisolone at FU (yes/no), n	10 (16.9)	7 (12.5)	3 (4.8)	0.13
Methotrexat at FU, n	10 (8.5)	9 (16.1)	1 (1.6)	**0.01**
Prednisolone during disease course, n	51 (86.4)	26 (92.9)	25 (80.6)	0.17
Methotrexat during disease course, n	30 (50.8)	18 (64.3)	12 (38.7)	**0.05**
Prednisolone medication time, months	31 (14.0–57.0)	24.5 (13.3–35.0)	48.0 (16.0–85.0)	**0.05**
Cumulative prednisolone total, g	7.90 (30.00–79.23)	6.14 (0.00–16.23)	9.68 (0.00–79.23)	**0.03**
Cumulative prednisolone 6 m after diagnosis^a^, g (*n* = 58/28/31)	2.76 (0.00–6.65)	2.89 (0.00–6.38)	2.61 (0.00–6.65)	0.60
Cumulative prednisolone 2 y after diagnosis, g	4.81 (0.00–0-16.50)	4.26 (0.00–9.80)	6.75 (0.00–16.49)	0.19
Cumulative prednisolone 6.5 y after diagnosis, g	7.35 (0.00–28.39)	6.14 (0.00–12.64)	9.43 (0.00–28.40)	0.18
Time from last prednisolone dose to FU, months	67.0 (0–162.0)	42.7 (0–67.8)	145.0 (0–253.0)	**0.002**

*FU* follow up, ^a^ *n* = 56, values are n (%) and median (range). *p*-values when comparing JDM <20y and JDM ≥20y

### Serum markers of bone and mineral metabolism in study participants

In all patients, reduced levels of 25(OH)VitD (Δ = 11 nmol/L) and higher levels of phosphate (Δ = 0.10 nmol/L) were found compared with controls (*p*-values =0.001 and 0.005) (Table 4). Also, total calcium and albumin levels were lower in patients than in controls (Δ = 0.04 nmol/L and 1.5 nmol/L, *p*-values = 0.004 and 0.002); however, insufficient levels of 25(OH)VitD and ionised calcium were comparable between patients and controls.

**Table 4:** Bone remodeling factors in JDM patients and controls

	Patients (***n*** = 55–58)	Controls (***n*** = 55–58)	***p***-values
CTX, ug/l	0.51 (0.34–0.97)	0.46 (0.30–0.96)	0.34
P1NP, ug/l	73.0 (46.8–318.0)	75.0 (52.8–352.0)	0.63
25-OH-VitD, nmol/l	54.0 (37.0–65.0)	65.0 (45.0–83.0)	**0.001**
Low 25(OH)VitD (< 37 nmol/l), n	13 (22.4)	4 (6.9)	0.08
Insufficient 25(OH)VitD (<20nmo/l), n	3 (5.5)	0 (0)	**0.02**
Calsium, nmol/l	2.29 (2.24–2.33)	2.33 (2.29–2.39)	**0.001**
iCalsium, nmol/l	1.25 (0.04)	1.25 (0.03)	0.83
Albumin, g/l	44.5 (2.3)	45.9 (2.3)	**0.002**
Alkaline phosphatase, U/l	71.0 (56.0–147.3)	72.00 (55.0–150.8)	0.97
Phosphate, nmol/l	1.23 (0.25)	1.13 (0.21)	**0.005**
PTH, pmol/l	3.52 (1.19)	3.53 (1.27)	0.95
E-SR, mm/h	5.0 (3.0–9.0)	4.0 (3.0–8.0)	0.06
hsCRP, mg/l	0.98 (0.28–2.6)	0.59 (0.23–1.27)	0.12
IP-10, pg/ml	1009 (911–1581)	969 (751–1187)	**0.04**
**Age < 20 y**	**(*n*** **= 25–28)**	**(n = 25–28)**	
CTX, ug/l	0.94 (0.55–1.39)	0.97 (0.49)1.22	0.73
P1NP, ug/l	326.0 (95.0–560.78)	397.0 (95.0–608.7)	0.77
25-OH-VitD, nmol/l	55.4 (19.0)	66.2 (24.4)	0.11
Low 25(OH)VitD (< 37 nmol/l), n	6 (21.4)	2 (7.1)	0.31
Insufficient 25-OH-VitD (<20 nmol/l), n	1 (3.7)	0 (0)	0.13
Calsium, nmol/l	2.3 (0.2)	2.3 (0.01)	0.18
iCalsium, nmol/l	1.26 (0.03)	1.26 (0.03)	0.75
Albumin, g/l	44.8 (2.1)	45.8 (2.1)	0.10
Alkaline phosphatase, U/l	149.5 (74.3–226.8)	152.5 (76.5–194.8)	0.60
Phosphate, nmol/l	1.35 (0.23)	1.23 (0.23)	**0.005**
PTH, pmol/l	3.55 (1.23)	3.32 (1.28)	0.50
E-SR, mm/h	4.50 (3.00–8.25)	4.50 (3.00–10.50)	0.44
hsCRP, mg/l	0.40 (0.24–2.44)	0.35 (0.19–1.27)	0.43
IP-10, pg/ml	982 (855–1479)	889 (732–1125)	0.14
**Age ≥ 20 y**	**(n = 28–30)**	**(n = 28–30)**	
CTX, ug/l	0.40 (0.24–0.49)	0.32 (0.23–0.41)	0.16
P1NP, ug/l	55.0 (41.8–59.0)	59.0 (42.8–68.0)	0.23
25(OH)VitD, nmol/l	47.3 (17.2)	65.3 (21.4)	**0.001**
Low 25(OH)VitD (< 0.37 nmol/l), n	7 (23.3)	2 (6.7)	0.15
Insufficient 25(OH)VitD (<20 nmol/l), n	2 (7.1)	0 (0)	0.07
Calsium, nmol/l	2.25 (0.11)	2.34 (0.09)	**0.002**
iCalsium, nmol/l	1.24 (0.04)	1.24 (0.03)	0.62
Albumin, g/l	44.3 (2.5)	46.0 (2.5)	**0.004**
Alkaline phosphatase, U/l	61.0 (49.0–73.3)	59.0 (50.0–70.5)	0.66
Phosphate,nmol/l	1.10 (0.22)	1.03 (0.17)	0.15
PTH, pmol/l	3.5 (1.17)	3.73 (1.44)	0.48
E-SR, mm/h	7.0 (3.0–8.5)	4.0 (3.0–8.0)	0.08
hsCRP, mg/l	1.07 (0.43–3.5)	0.63 (0.33–1.27)	0.15
IP-10, pg/ml	1066 (940–1593)	1041 (764–1201)	0.14

*CTX* C-terminal collagen crosslinks, *P1NP* pro-collagen type 1 N-terminal pro-peptide, *25-OH-VitD* 25-hydroxyvitamin D, insufficient 25(−OH)-VitD <20 nmol/l; Low 25(OH)VitD, *n* < 37 nmol/l; iCalsium: ionized calcium, *PTH* parathyroid hormone, *E-SR* erythrocyte sedimentation rate, *hsCRP* high sensitive C-reactive protein, *IP-10* Interferon gamma-induced protein 10. Values are mean (SD) or median (IQR). *P*-values when comparing patients and controls using independent samples t-test or chi-square for nonparametric values

In study participants <20y, phosphate levels were higher in patients compared with the respective controls (Δ = 0.12 nmol/L, *p*-value =0.005). There were no significant differences in levels of any bone remodelling factors including CTX, P1NP and PTH between patients and controls nor between the age groups </≥20y (Table 4).

### Associations between BMD Z-scores and prednisolone use and dosage in patients

For all patients, we found a negative correlation between prednisolone use at follow-up (yes/no) and the Z-score of spine (Table 5). In patients <20y, all prednisolone variables (use at follow-up, use in months and cumulative dosage) correlated negatively with the BMD Z-score for spine (Table 5). In patients ≥20y, there were positive correlations between prednisolone use in months and the Z-score for WB (Table 5).

**Table 5:** Associations between BDM and BMD Z-score and: prednisolone and bone remodeling factors in JDM patients and controls, younger and older than 20 years

	Patients	Controls	Patients	Controls	Patients	Controls
	Total	Total	Age < 20y	Age < 20y	Age ≥ 20y	Age ≥ 20y
**BMD Z-score, Whole body**						
Prednisolone use at FU	−0.26	NA	−0.26	NA	−0.20	NA
Prednisolone use (month)	0.18	NA	−0.36	NA	0.43*	NA
PTH	− 0.30*	0.01	− 0.29	0.15	− 0.33	− 0.12
IP-10	− 0.34*	0.16	− 0.38	0.14	− 0.38*	0.25
hsCRP	−0.15	0.234	−0.21	0.54**	−0.22	0.07
**BMD Z-score, Lumbar spine**						
Prednisolone use at FU	−0.41*	NA	−0.45*	NA	−0.34	NA
Prednisolone use (month)	0.01	NA	−0.40*	NA	0.18	NA
Cumulative prednisolone	0.00	NA	−0.48*	NA	0.15	NA
IP-10	−0.21	0.20	−0.42*	0.10	−0.19	0.19
hsCRP	−0.24	0.19	−0.19	0.39*	−0.40*	0.03

*FU* follow up, *25-OH-VitD* 25-hydroxyvitamin D, *IP-10* interferon gamma-induced protein 10, *hsCRP* high sensitive C-reactive protein, *NA* not applicable All values are Spearmans correlation or point bi-serial correlations when appropriate, * *p* < 0.05, **p < 0

### Associations between levels of bone remodelling factors and BMD Z-score in study participants

In all patients, we found levels of PTH and IP-10 to correlate negatively with the BMD Z-score in WB (*p*-values =0.01 and 0.03) (Table 5). In patients <20y levels of IP-10 correlated negatively with the BMD Z-score for spine. We also found a positive correlation between levels of hsCRP and the BMD Z-score in WB in controls <20y. In patients ≥20y: levels of IP-10 were negatively associated with the BMD Z-score for WB. Further, the ESR and hsCRP were negatively associated with the BMD Z-score for spine in patients ≥20y (Table 5).

### Correlates of BMD Z-scores in patients

In multivariate linear regression models, the BMD Z-scores for the WB and spine were used as dependent variables. The age at diagnosis, age ≥ 20y at follow-up, prednisolone use at follow-up (yes/no), and low levels of 25(OH)VitD, IP-10 and hsCRP were treated as independent variables. We found age ≥ 20y to be correlated to the BMD Z-score in WB (*p*-value =0.05), and prednisolone use at follow-up (yes/no) was associated with the BMD Z-score in spine (*p*-value =0.007) (Supplementary Table 1).

## Discussion

Data on bone health in JDM after long-term follow-up is lacking. We found higher prevalence of reduced BMD Z-scores in both WB and spine in patients than in controls and bone density was differently affected in patients <20y vs. ≥20y. In patients <20y, bone density of WB and spine were lower compared with controls and was negatively associated with prednisolone dose. In patients ≥20y, only bone density in the forearm was lower compared with controls and BMD Z-score for spine correlated negatively with inflammatory parameters. The biomarker of JDM activity, IP-10 was associated with lower BMD Z-scores in both age groups. In all patients, prednisolone use at follow-up (yes/no) and age ≥ 20y were independent correlates of BMD Z-scores in WB and spine, respectively.

Our findings of more frequent reduced BMD Z-scores for WB and spine in all patients vs controls are in line with a study from our centre, which examined children and adults with juvenile-onset SLE (jSLE) [26]. By including both children and adults, we had the unique opportunity to evaluate whether and how BMD was affected early and late in JDM disease course. Due to differences in DXA acquisition regions and expected differences in bone mineralisation status between children and adults [30], we discuss the findings of patients <20y and ≥ 20y separately.

In patients <20y, BMD and BMD Z-scores in both WB and spine were lower compared with respective controls. Similar results have been found in both untreated JDM [13] and after 0.2–8.3y [12, 13]. Our finding of reduced BMD Z-scores of spine in 36% of our patients <20y (after median 6.5y disease duration) is comparable with frequencies found in children with chronic rheumatic diseases (including JDM) (25–70%) after variable disease durations [2, 14, 16, 26, 27]. Patients ≥20y had lower BMD and BMD Z-scores of the forearm than controls, and 42% of these patients had reduced BMD Z-scores. This is line with other studies of adult patients with juvenile-onset autoimmune diseases including juvenile idiopathic arthritis (50%) [31] and jSLE (≈ 30%) [26]. Possible explanatory factors for reduced bone density in our patients are discussed below.

Prednisolone (months used, use at follow-up and cumulative doses) was associated with lower bone mass in the spine, especially in patients <20y. It is well established that prednisolone influences bone modelling negatively in children [1], especially of the spine [32]. However, studies addressing how prednisolone treatment in JDM affects BMD are conflicting as some find negative correlations between corticosteroid treatment and bone density [2, 15] while others do not [14] . In addition, the effects of prednisolone are likely to be duration- and dose-dependent [1].

Since the ≥20y-group had fewer pathological BMD findings, one could speculate that they had received less prednisolone than the <20y-group. However, after 6.5 years (which is median disease duration for patients <20y), patients ≥20y had 1.5 times higher cumulative prednisolone dose (borderline significance) than those <20y. Also, age ≥ 20y was identified as an independent correlate of low BMD Z-scores for WB. The association between higher BMD Z-scores for WB and prednisolone use in months in patients ≥20y is surprising. However, patients ≥20 used prednisolone > 3 times longer during disease course (not significant) and had longer time off prednisolone compared to patients <20y. It might be that bone density may improve and bone health may reconstitute after corticosteroid discontinuation [33].

Here for the first time we report levels of CTX and P1NP in JDM patients; both were comparable between patients and controls. It is known that glucocorticoid treatment negatively affects both CTX and P1NP [11, 34]. Hence, in our patients normal CTX and P1NP levels might be mirroring differences in treatment plans, and the aforementioned reconstituted bone health, rather than an effect from prednisolone.

The biomarker of JDM, IP-10 [29], correlated negatively with BMD Z-scores in both age groups. Downregulation of IP-10 has been found to decrease osteoclast differentiation and thereby prevent bone degradation [35]. However, to our knowledge, the effect of IP-10 upon BMD has not been examined in other autoimmune inflammatory diseases. Interestingly, studying the same cohort, we have previously found an association between higher IP-10 and pulmonary involvement [36], which indicates a multi-organ role of this cytokine in JDM that should be studied further. Additionally, the inflammatory markers ESR and hsCRP were associated with low BMD Z-scores of spine in patients ≥20y [37], which is in line with findings in both children and adults with autoimmune rheumatic diseases [12, 16, 31]. Also, levels of CRP might predict impaired bone density in general [38].

All patients and patients ≥20y had lower vitamin D levels than the controls. Low vitamin D contributes to a lower BMD by increasing bone remodelling [39]. Data on vitamin D levels in IIM including JDM is conflicting [15, 40, 41]. Vitamin D levels might be reduced in JDM patients [38, 39] due to patients’ photosensitivity [40], involuntarily reduction in sun-exposure and insufficient dietary vitamin D. Regrettably, we have no data on dietary vitamin D intake.

Strengths of our study include that 95% of all identified and tracked JDM patients participated in the study; our results are thus less biased toward serious cases compared to other outcome studies [42]. Since our age- and sex-matched controls were randomly drawn from the Norwegian registry, we believe they reflect the general population. Also, the external validity of our results is supported by the finding of MDI assessed osteoporosis in 5 (8.5%) patients [17], which is in line with other studies [18, 19]. There are some limitations to our study other than those already discussed: we did not adjust for multiple testing due to the hypothesis generating nature of our study. We used < 20/>20y as age-cut-off, which does not necessarily correlate with sexual maturation and developmental stages of importance to bone maturation. Tanner stage was only reported from a limited number of patients <20y and hence not included in the study. Also, volumetric BMD and skeletal age were not assessed, leaving us unable to discuss these measures as explanatory factors. We are also limited by the lack of prospective data on disease activity.

## Conclusion

After long-term follow-up, bone density in JDM children and adults are differently affected. Compared with controls, both children and adolescents with JDM show reductions in BMD and BMD Z-scores in both WB and spine. The shown association between impaired BMD Z-scores and prednisolone use at follow-up (yes/no), age > 20y and higher levels of inflammatory markers confirms already established associations, while the association with the JDM biomarker IP-10 found in adult patients is novel and should be studied further.

## Supplementary Information

**Additional file 1: Supplementary Table 1**. Correlates of BMD Z-score whole body and lumbar spine in patients.

## Data Availability

Due to ethical concerns, supporting data cannot be made openly available. The datasets generated during and/or analyzed during the current study are not publicly available due to safeguarding by the EU General Data Protection Regulation and Norwegian law.

## Abbreviations

JDM: Juvenile dermatomyositis
BMD: Bone mineral density
DXA: Dual-energy X-ray absorptiometry
25(OH)VitD: 25-hydroxyvitamin D
PTH: parathyroid hormone
P1NP: Procollagen type 1 N-terminal propeptide
CTX: C-terminal telopeptide of type 1 collagen
SLE: Systemic lupus erythematosus
MDI: Myositis damage index
OUS: Oslo university hospital
DAS: Disease activity score
PCS: physical component summary scale
SF-36: Short Form 36 health survey
WB: Whole body
IP-10: Interferon gamma-induced protein 10
ESR: Erythrocyte sedimentation rate
hsCRP: high-sensitivity C-reactive protein
jSLE: Juvenile-onset SLE
