# Supplementary material for: Bone mineral density and explanatory factors in children and adults with juvenile dermatomyositis at long term follow-up; a cross sectional study
Source: Pediatr Rheumatol Online J. 2021 Apr 26;19:56. doi: 10.1186/s12969-021-00543-z (PMC8077908; doi:10.1186/s12969-021-00543-z)
Supplement: Supplementary file 1 — Additional file 1: Supplementary Table 1. Correlates of BMD Z-score whole body and lumbar spine in patients. [file 12969_2021_543_MOESM1_ESM.docx]

Supplementary table 1. Correlates of BMD Z-score whole body and lumbar spine in patients

| Dependent variables | Independent  variables | | |  | Univariate  analysis | | Multivariate  analysis | |  |
| --- | --- | --- | --- | --- | --- | --- | --- | --- | --- |
|  |  |  | | | B | 95% CI | B | 95% CI | p |
| Z-score BMD, whole body | | |  | |  |  |  |  |  |
|  | Age at diagnosis | | | | 0.21 | (-0.01, 0.04) | 0.00 |  |  |
|  | Age ≥20y |  | | | 0.61 | (0.06, 1.15) | 0.47 | (0.01, 1.20) | 0.045 |
|  | Prednisolone use at FU | | | | -0.78 | (-1.51, -0.46) | -0.48 |  |  |
|  | Insufficient VitaminD | | | | 0.32 | (-0.70, 0.73) | -0.08 |  |  |
|  | IP-10 |  | | | 0.00 | (0.00, 0.00) | 0.00 |  |  |
|  | hsCRP |  | | | -0.05 | (-0.14, 0.04) | -0.04 |  |  |
| Z-score BMD, lumbar spine | | | | |  |  |  |  |  |
|  | Age at diagnosis | | | | 0.02 | (-0.01, 0.04) | 0.00 |  |  |
|  | Age ≥20y |  | | | 0.45 | (-0.17, 1.08) | 0.26 |  |  |
|  | Prednisolone use at FU | | | | -1.23 | (- 2.01, -0.45) | -1.13 | (-1.93, -0.32) | 0.007 |
|  | Insufficient VitaminD | | | | 0.25 | (-0.52, 1.02) | 0.16 |  |  |
|  | IP-10 |  | | | 0.00 | (0.00, 0.00) | 0.00 |  |  |
|  | hsCRP |  | | | -0.08 | (-0.18, 0.02) | 0.05 |  | |

All variables assessed at follow-up if not otherwise stated; Results from the final model of multiple linear regression analysis (forward regression method), B: unstandardized beta coefficient using univariate linear regression. FU: follow up, IP-10: Interferon gamma-induced protein 10, hsCRP: high sensitive C-reactive protein
